# Autoimmune hepatitis or drug‐induced autoimmune hepatitis following Covid‐19 vaccination?

**DOI:** 10.1111/liv.15224

**Published:** 2022-03-11

**Authors:** Federica Fimiano, Daphne D’Amato, Alessandro Gambella, Alfredo Marzano, Giorgio M. Saracco, Anna Morgando

**Affiliations:** ^1^ Gastroenterology Unit, Città della Salute e della Scienza Turin Italy; ^2^ Anatomopathology Department, Città della Salute e della Scienza Turin Italy

**Keywords:** autoimmune hepatitis, Covid‐19 vaccine, drug‐induced autoimmune hepatitis

**Abbreviations**
AIH: autoimmune hepatitis
AST: aspartate aminotransferase
ALT: alanine aminotransferase
ALP: alkaline phosphatase
GGT: gamma‐glutamyl transferase
CT: computerized tomography
MRI: magnetic resonance imaging
IgG: immunoglobulins G
ANA: anti‐nuclear antibodies
AMA: anti‐mitochondrial antibodies
ASMA: anti‐smooth muscle antibodies
LKM‐1: anti‐liver‐kidney microsomal antibodies
ANCA: and anti‐neutrophil cytoplasmic antibodies
UNL: upper normal level
DI‐AIH: drug‐induced autoimmune hepatitis

Several cases of autoimmune hepatitis (AIH) after coronavirus disease 2019 (Covid‐19) vaccination have been recently reported.^1^, ^2^, ^3^, ^4^ However, no data to prove a casual relation between vaccine and AIH is still available. More recently, a case of acute immune‐mediated hepatitis after severe acute respiratory syndrome (Sars Cov‐2) Moderna Vaccine has been reported.^5^ This is a hot topic since these vaccines are very recent, the worldwide population is going to be vaccinated, and the post‐marketing phase 4 for these new drugs is still ongoing. Here, is the case of a 63 years‐old Caucasian female patient. In her medical history postmenopausal hypothyroidism, familiarity with autoimmune conditions (sister affected by the coeliac disease) were reported. The patient completed BNT162b2 mRNA (Pfizer‐BioNTech) Covid 19 vaccination on 15 July. She did not experience Covid‐19 disease.

On 7 September, the patient was admitted to the emergency department with abdominal pain, nausea, associated with hyperchromic urines, jaundice and hypoechoic stools. Biochemical tests showed aspartate aminotransferase (AST) 1625 UI/L, alanine aminotransferase (ALT) 1778 UI/L, alkaline phosphatase (ALP) 273, gamma‐glutamyl transferase (GGT) 419 UI/L, total bilirubin/direct bilirubin 18.6/14.2 mg/dl. She underwent abdominal ultrasound, abdominal computerized tomography (CT) scan and magnetic resonance imaging (MRI), which showed a normal liver, with no signs of chronic liver injury, no signs of gallstones nor alteration of the biliary tree. A wide serological hepatological screening was performed: acute viral hepatitis (hepatitis A, hepatitis B, hepatitis C, hepatitis E, cytomegalovirus, Epstein–Barr virus, herpes simplex virus 1/2, human herpesvirus 6), hemochromatosis, Wilson disease and toxics were excluded.

At the autoimmune screening, there was an anti‐thyroglobulin antibodies positivity, immunoglobulins G (IgG) were slightly increased (17.60 g/L, normal values 7–16 g/L), anti‐nuclear antibodies (ANA), anti‐mitochondrial antibodies (AMA), anti‐smooth muscle antibodies (ASMA), anti‐liver‐kidney microsomal antibodies (LKM‐1) and anti‐neutrophil cytoplasmic antibodies (ANCA) were negative. Notably, the antibodies anti‐Sars‐CoV 2 spike protein was higher than 35 000 BAU/ml, more than 1000 times the upper normal level (UNL).

During the hospitalization, because of bilirubin and transaminases levels were stably increased, the autoimmunity was negative and the IgG title was mildly upper of normal she underwent liver biopsy on 15 September. Histology demonstrated severe acute hepatitis with an intense portal/periportal and lobular plasma cell‐rich inflammation, interface activity and focal confluent necrosis. Centrilobular congestion, hepatocellular ballooning and scattered apoptotic bodies were also observed (Figure 1). Confirming the AIH‐like features, methylprednisolone at 1 mg/kg/daily was started; after that, a slow but constant reduction of transaminases and bilirubin was observed. Azathioprine 50 mg daily was imbricated, obtaining a further improvement of the liver tests.

**FIGURE 1:**
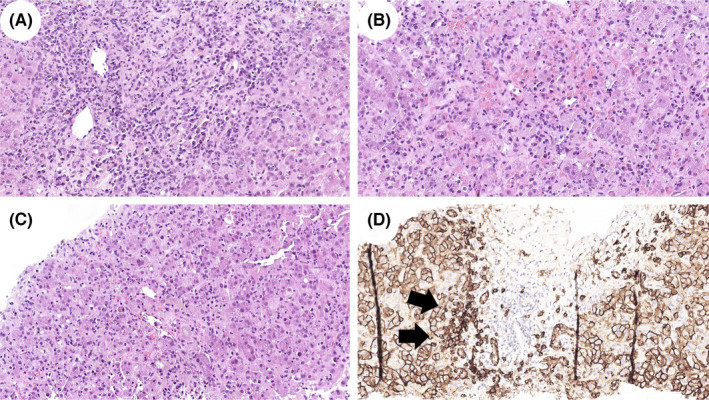
Acute hepatitis with lymphoplasmacytic‐rich inflammatory infiltrate. The biopsy showed a severe hepatic injury with intense portal/periportal lymphoplasmacytic infiltrate and interface hepatitis (A; haematoxylin and eosin, original magnification 200×). Lobular inflammation, centrilobular congestion, and confluent necrosis were also noticed (B, C; haematoxylin and eosin, original magnification 200×). Immunohistochemical staining for CD138 (D; original magnification 200×) highlighted plasma cells infiltrate with diffuse involvement of portal tract interface (black arrows)

Notably, the latency period between the vaccination and the hospitalization is longer compared to other cases previously reported.^1^, ^2^, ^3^, ^4^ Though, the onset of AIH may have been earlier and subclinical, therefore, diagnosed only when needing medical attention.

Moreover, there are no confounding factors (such as other medication, toxics or pregnancy) regarding the medical history of the hereby presented patient.

As known, AIH could be triggered by some viruses, including COVID 19,^6^ by vaccination^7^ and by drugs^8^ and liver injury can be toxic through a direct or immunomodulated mechanism. In the latter case, we talk about drug‐induced autoimmune hepatitis (DI‐AIH) that is different from classic AIH because of no recurrence after glucocorticoids withdrawal. It is still unclear whether we are facing a drug‐induced acute hepatitis with autoimmune features, or a ‘real’ AIH triggered by the covid vaccines.

It might be interesting to know if the patients reported in the previously published cases relapsed after glucocorticoids withdrawal. In fact, there is only one case reported in the literature, of immune‐mediated hepatitis occurring after the first dose of Moderna vaccine, and relapse after the second dose.^5^

In view of 3rd dose of the Covid vaccine, discerning between a classic AIH and DI‐AIH is essential in order to avoid hepatitis exacerbations which may escalate to fulminant hepatitis.

Therefore, clinical studies are necessary to draw a conclusion on a possible cause‐effect relationship and larger epidemiological studies are needed to investigate whether the incidence of AIH has significantly increased owing to the global vaccination against Covid 19.

This report does not intend to create public concern regarding the safety of the Covid 19 vaccine.

## CONFLICT OF INTEREST

The authors do not have any disclosures to report.

## CODE AVAILABILITY

Not applicable.
